# RNA-Seq of Guar (*Cyamopsis tetragonoloba*, L. Taub.) Leaves: *De novo* Transcriptome Assembly, Functional Annotation and Development of Genomic Resources

**DOI:** 10.3389/fpls.2017.00091

**Published:** 2017-02-02

**Authors:** Umesh K. Tanwar, Vikas Pruthi, Gursharn S. Randhawa

**Affiliations:** Department of Biotechnology, Indian Institute of Technology RoorkeeRoorkee, India

**Keywords:** next generation sequencing, transcriptome analysis, molecular markers, simple sequence repeats, single nucleotide polymorphisms

## Abstract

Genetic improvement in industrially important guar (*Cyamopsis tetragonoloba*, L. Taub.) crop has been hindered due to the lack of sufficient genomic or transcriptomic resources. In this study, RNA-Seq technology was employed to characterize the transcriptome of leaf tissues from two guar varieties, namely, M-83 and RGC-1066. Approximately 30 million high-quality pair-end reads of each variety generated by Illumina HiSeq platform were used for *de novo* assembly by Trinity program. A total of 62,146 non-redundant unigenes with an average length of 679 bp were obtained. The quality assessment of assembled unigenes revealed 87.50% of complete and 97.18% partial core eukaryotic genes (CEGs). Sequence similarity analyses and annotation of the unigenes against non-redundant protein (Nr) and Gene Ontology (GO) databases identified 175,882 GO annotations. A total of 11,308 guar unigenes were annotated with various enzyme codes (EC) and categorized in six categories with 55 subclasses. The annotation of biochemical pathways resulted in a total of 11,971 unigenes assigned with 145 KEGG maps and 1759 enzyme codes. The species distribution analysis of the unigenes showed highest similarity with *Glycine max* genes. A total of 5773 potential simple sequence repeats (SSRs) and 3594 high-quality single nucleotide polymorphisms (SNPs) were identified. Out of 20 randomly selected SSRs for wet laboratory validation, 13 showed consistent PCR amplification in both guar varieties. *In silico* studies identified 145 polymorphic SSR markers in two varieties. To the best of our knowledge, this is the first report on transcriptome analysis and SNPs identification in guar till date.

## Introduction

Guar [*Cyamopsis tetragonoloba*, L. Taub.], also known as clusterbean, is an annual drought-tolerant legume crop belonging to the family Leguminosae. It is grown mainly in semiarid regions of India, Pakistan, and the United States. Guar has been traditionally used as a forage, green manure and vegetable crop (Dwivedi et al., 1995). In recent times, it has attained the status of an economically important crop because of the gum contained in endosperm of its seeds. Guar gum contains about 90% galactomannan and it is one of the most cost-effective natural thickeners (Dhugga et al., 2004). It is used in textile, paper, petroleum, explosives, cosmetics, and pharmaceutical industries (Yadav et al., 2013). Additionally, guar gum is used in the treatment of diarrhea, irritable bowel syndrome, diabetics, and high cholesterol (Slavin and Greenberg, 2003; Giannini et al., 2006; Butt et al., 2007). Therefore, the demand for guar has increased globally in recent years, leading to its introduction in several countries including South Africa, Australia and Brazil having varied climates and seasons (Undersander et al., 1991). As a result, there is a need to develop, through breeding programs, improved guar varieties for wide range of climatic conditions.

Molecular markers have been found useful in breeding programs involving marker-assisted selection and their use has reduced time and effort for developing improved varieties (Kesawat and Kumar, 2009). These markers are a tool to detect genetic polymorphism at specific loci and whole-genome level as they facilitate marker-based gene tagging, genetic mapping, map-based cloning of agronomically important genes, genetic diversity studies, and phylogenetic analysis (Morgante et al., 2002; Kesawat and Kumar, 2009). Five molecular markers, namely, random amplified polymorphic DNA (RAPD), ribosomal DNA (rDNA), inter simple sequence repeat (ISSR), simple sequence repeat (SSR) and sequence characterized amplified region (SCAR) have been used in the study of molecular diversity in guar (Punia et al., 2009; Pathak et al., 2010, 2011; Kuravadi et al., 2013, 2014; Sharma et al., 2014; Kumar et al., 2016). Among the various molecular markers, SSR and single nucleotide polymorphism (SNP) markers are considered to be very important in genetic and plant breeding applications (Hiremath et al., 2012). However, limited number of SSR markers are available in guar (Kuravadi et al., 2014; Kumar et al., 2016) and no SNPs have yet been reported in this crop.

Next generation sequencing (NGS) offers novel opportunities in functional genomics, gene identification and development of molecular markers in non-model plants (Wang et al., 2009). The massive parallel sequencing of RNA (RNA-Seq or transcriptome profiling) is a powerful tool for transcription profiling, providing a rapid access to a large collection of expressed sequences (transcriptome). This sequencing approach is more efficient than the traditional expressed sequence tag (EST) sequencing. RNA-Seq technology has been successfully applied in several organisms including model and non-model plants (Mortazavi et al., 2008; Nagalakshmi et al., 2008; Parchman et al., 2010; Wang et al., 2014). This technology can be used as a cost-effective source for the development of molecular markers such as SSRs and SNPs (Wang et al., 2011, 2014). These transcriptome-derived markers are expected to show greater transferability among closely related species than that of the genomic markers because of their presence in more-conserved transcribed regions of the genome (Cordeiro et al., 2001). These markers can also be used for comparative mapping and evolutionary studies (Varshney et al., 2005b).

At present complete genome sequences of five legumes, namely, soybean, *Lotus, Medicago*, pigeonpea, and chickpea, are available (Sato et al., 2008; Schmutz et al., 2010; Young et al., 2011; Varshney et al., 2012, 2013; Jain et al., 2013). Guar genome sequencing and transcriptome analysis of guar have not been yet done. Only 16,476 ESTs from developing guar embryos are available in National Center for Biotechnology Information (NCBI) database. The breeding programs in guar have been hindered due to the limited availability of genomic resources in this crop. The development of genomic resources for guar is needed to support molecular genetics research at different levels. Therefore, the present study was undertaken to develop genomic resources based on the sequencing of cDNA pools from leaf tissues of two guar varieties (M-83 and RGC-1066) which were selected because of their contrasting characteristics.

## Materials and methods

### Plant material and transcriptome sequencing

The seeds of two guar varieties, namely, M-83 and RGC-1066, were obtained from Rajasthan Agricultural Research Institute, Durgapura, Jaipur (India). The variety M-83 has glabrous leaf surface, white flower color and it is a vegetable variety. The variety RGC-1066 has hairy leaf surface, purple flower color and is a commercial variety for gum production. The plants were grown in field conditions at Indian Institute of Technology Roorkee, India and healthy leaves were collected from 3-week-old plants. The sequencing of leaf transcriptome was outsourced to SciGenome Labs Pvt. Ltd., Cochin (India). Three technical and three biological replicates were used for library preparation and RNAseq. Total RNA from plant leaves of each variety was extracted by using SIGMA SpectrumTM Plant Total RNA Kit (Sigma-Aldrich, USA) and cDNA library of each variety was prepared by the procedure described in Illumina's TruSeq® RNA sample preparation guide (Illumina, Inc., USA). The sequencing of each cDNA library was carried out on an Illumina HiSeq 2500 machine to get pair-end sequence reads of 100 bp length. The raw data in FASTQ format was obtained from the company.

### *De novo* transcriptome assembly of guar leaf

The raw reads of leaf transcriptome of each guar variety were processed for quality control by FastQC version 0.11.4 software (Andrews, 2010). The adaptor sequences and low quality reads with ambiguous sequences “N” were removed to obtain the clean reads. The read orientation based pooling of the clean reads from both varieties was carried out. The pooled clean reads were uploaded to Transcriptomes User-Friendly Analysis (TRUFA) web server for cluster computing for *de novo* transcriptome assembly (Kornobis et al., 2015). The Trinity program (Grabherr et al., 2011) was employed for assembling the clean reads to obtain the unigene contigs. For the *de novo* transcriptome assembly, *k-mer* size was set as 25 and default values were used for other parameters. The assembled transcripts were clustered by the CD-HIT version 4.5.4 tool (Li and Godzik, 2006) with sequence identity threshold 0.95 to remove redundant transcripts. The quality check of the transcriptome assembly was done by assessing the presence of 248 ultra-conserved core eukaryotic genes (CEGs) in the assembly by Core Eukaryotic Genes Mapping Approach (CEGMA) computational method (Parra et al., 2007, 2009).

### Functional annotation of guar leaf transcriptome

Functional annotations were done by comparison of the sequences of clustered assembly with the public databases. The sequence similarity search of unitranscripts was carried out by BLASTX tool (Altschul et al., 1997). Homologs of the assembled unigenes were searched in the NCBI non-redundant protein (Nr), UniProt Reference Clusters (UniRef; Suzek et al., 2015) and Pfam (Finn et al., 2014) databases using default parameters. The BLAST+ (Camacho et al., 2009) results against the Nr database were imported to Blast2GO suite (Conesa et al., 2005) for mapping and retrieving Gene Ontology (GO) and unique enzyme code (EC) annotations of assembled unigenes. The retrieved GO terms were allocated to query sequences and the genes present in the transcriptome were classified into cellular component, molecular function and biological process categories. The WEGO tool (Ye et al., 2006) was used for functional classification and graphical representation of GO terms at macro level. The assembled unigenes were further annotated against the Kyoto Encyclopedia of Genes and Genomes (KEGG) metabolic pathways database (Kanehisa and Goto, 2000). The comparison of the assembled unigenes with the most closely related species was carried out by TRAPID online tool (Van Bel et al., 2013) with similarity search *E*-value 10e-5.

### Mining of simple sequence repeats (SSRs) of guar transcriptome

The mining of SSRs was done by searching six repeat motifs (mono-, di-, tri-, tetra-, penta-, and hexanucleotides) using the PERL script MIcroSAtellite (MISA) tool (Thiel et al., 2003). The following default definements (unit size/minimum number of repeats) were set in MISA for microsatellites: (1/10) (2/6) (3/5) (4/5) (5/5) (6/5). All motifs containing continuous uninterrupted repeats were classified as perfect and the motifs having two or more classes of repeats were classified as compound microsatellites. Maximal numbers of bases interrupting 2 SSRs in a compound microsatellite were set to 100.

### Validation of SSR markers

Twenty SSR markers representing all the motif types (except mononucleotide repeats) were selected randomly for wet laboratory validation. The primers were designed by Primer3 tool (Koressaar and Remm, 2007; Untergasser et al., 2012). The DNA was extracted by CTAB method (Doyle and Doyle, 1990) with slight modifications from the healthy leaves of field grown guar plants of each variety. The quality of extracted DNA was assessed by gel electrophoresis on 0.8% agarose gel. The isolated DNA was quantified by measuring the absorbance at 260 nm in a UV-visible Varian spectrophotometer, model Cary 100 and diluted with TE buffer to ~100 ng/μl. Polymerase chain reaction (PCR) was carried out in a Mastercycler gradient programmable thermal cycler (Eppendorf). PCR amplified products were electrophoresed on 8% PAGE gels and visualized under white light by silver staining. A 100 bp DNA ladder was used as a molecular marker to determine the approximate size of the fragments. The gel was documented in the gel documentation unit (Bio-Rad).

### *In silico* analysis of SSR polymorphism

The reads of each variety were mapped to the assembly using Bowtie2 version 2.2.6 (Langmead and Salzberg, 2012) software to obtain the sorted transcripts binary version of SAM files (BAM). *In silico* identification of SSR polymorphism was carried out using Integrative Genome Viewer (IGV 2.3) software (Robinson et al., 2011; Thorvaldsdóttir et al., 2013). The pairwise alignment of the sorted transcripts of both varieties was done against the assembly using IGV 2.3 software and the alignment was inspected manually to identify the SSR differences in guar varieties M-83 and RGC-1066.

### Detection of single nucleotide polymorphisms (SNPs)

The reads of each guar variety were aligned against the assembled unigenes by Bowtie2 version 2.2.6 (Langmead and Salzberg, 2012) software to obtain the sorted transcripts (BAM files) for each variety. The detection of SNPs was carried out by SAMtools 1.3 (Li et al., 2009) variant calling programms in Integrated SNP Mining and Utilization (ISMU) pipeline (Azam et al., 2014). The *de novo* assembly was used as a reference for SNP calling. A position was called a putative SNP if any variety had a different allele against the reference. The putative SNPs were further filtered for the homozygous allele types with a minimum read depth of 5 in each variety.

## Results

### RNA-seq and *de novo* transcriptome assembly of guar leaf

**Figure 1:**
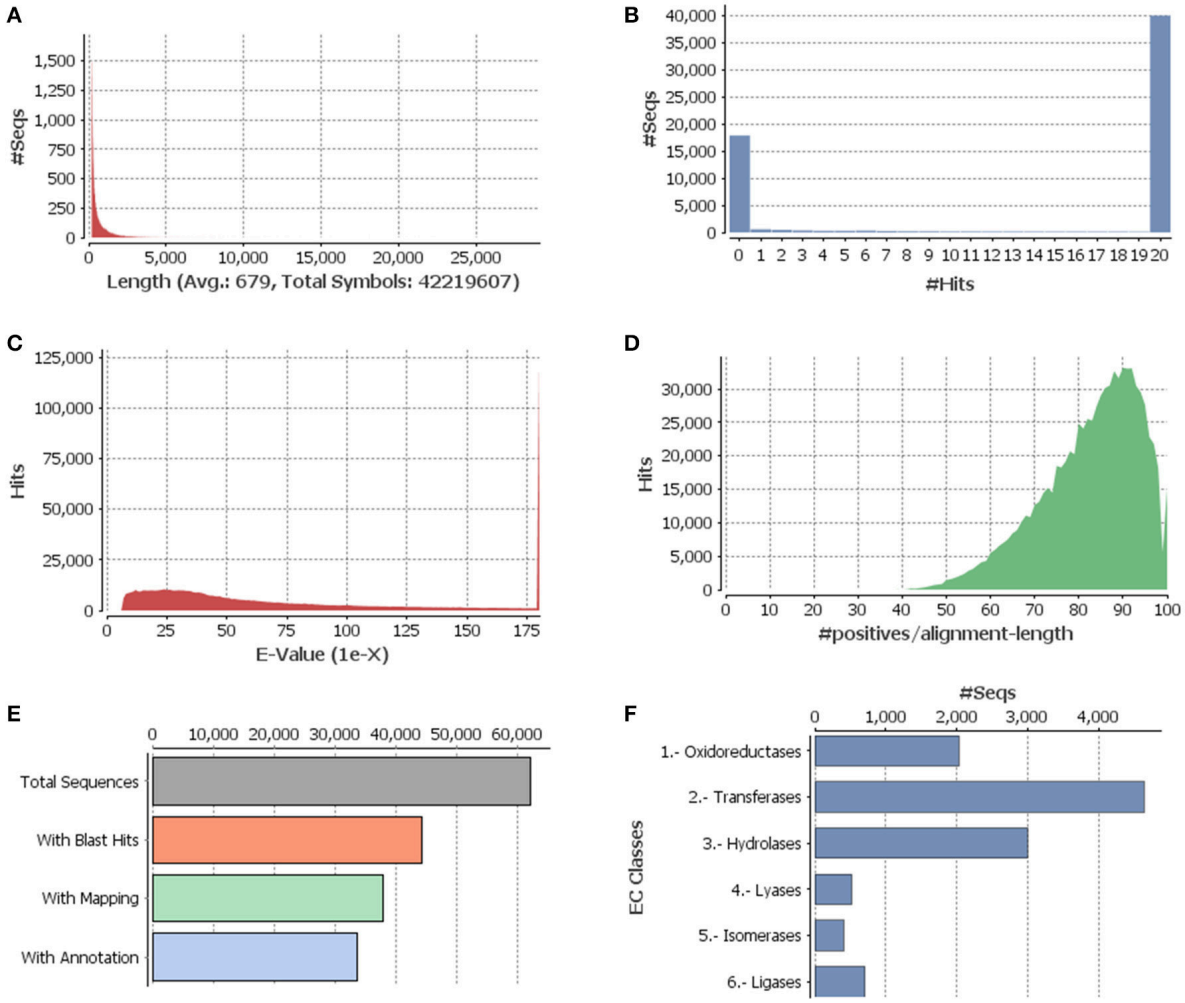
**Functional annotation of guar leaf transcriptome. (A)** Sequence distribution, **(B)** Blast hit distribution, **(C)** *E*-value distribution of BLAST hits for each unique sequence against the Nr database, **(D)** Similarity distributions of the top BLAST hits for each sequence against the Nr database, **(E)** Distribution of Blast2GO three step processes including BLASTX, mapping and annotation, and **(F)** Enzyme code distributions.

The Illumina HiSeq sequencing platform generated 28,688,024 and 33,018,878 raw pair-end reads for the guar varieties M-83 and RGC-1066, respectively. The sequence reads have been submitted to NCBI-SRA database (Temporary Submission ID: SUB1380346). The mean read quality (Phred Score) and % Q > 30 of these reads were ~35 and 90, respectively. The average read length was 100 bp for each variety (Supplementary Table S1). The cleaning and read orientation based pooling of the reads of both varieties resulted in a total of 42,777,004 (R1) and 59,940,380 (R2) clean reads with an average length of 88 bp. The *de novo* assembly of all the clean reads by Trinity program (Grabherr et al., 2011) generated 79,355 contigs. The clustering of assembled sequences using CD-HIT version 4.5.4 tool (Li and Godzik, 2006) gave 62,146 unigenes having 679 bp average length and 1035 bp N50 value (Table 1). The shortest and longest unigenes were 201 and 29,056 bp, respectively. The length of 37,352 unigenes was <500 bp whereas 24,794 unigenes were having the length of more than 500 bp size. A total of 11,593 unigenes were over 1000 bp and 237 unigenes were over 5000 bp (Figure 1A).

**Table 1:** **Statistics of *de novo* assembly of guar leaf transcriptome**.

**Characteristic**	**Details**
Total number of contigs	62,146
Min length	201
Max length	29,056
Average length	679.36
Standard deviation	792.86
Median length	394.0
Total bases in contigs	42,219,607
Number of contigs <500 pb	37,352
Number of contigs ≥ 500 pb	24,794
Number of contigs ≥ 1000 pb	11,593
Number of contigs ≥ 2000 pb	3292
Number of contigs ≥ 5000 pb	237
Number of contigs ≥ 10000 pb	38
N50	1035.0
Contigs in N50	11,028
GC content	43.68%

The clean reads were mapped to the assembled unigenes to assess the quality of assembly. The overall alignment rate was 71%. Among the mapped reads 74% reads could uniquely map to the unigenes, while 11% reads could map to multiple locations on unigenes. In addition, analysis of the presence of CEGs revealed that the assembly had 87.50% of complete and 97.18% partial CEGs against the 248 CEGs as reference (Table 2).

**Table 2:** **Statistics of CEGMA results^#^ of guar leaf transcriptome assembly**.

	**Prots**	**%Completeness**	**Total**	**Average**	**%Ortho**
Complete	217	87.50	490	2.26	70.51
Group 1	55	83.33	131	2.38	78.18
Group 2	45	80.36	101	2.24	73.33
Group 3	55	90.16	129	2.35	67.27
Group 4	62	95.38	129	2.08	64.52
Partial	241	97.18	661	2.74	80.50
Group 1	64	96.97	176	2.75	82.81
Group 2	54	96.43	158	2.93	85.19
Group 3	58	95.08	164	2.83	72.41
Group 4	65	100.00	163	2.51	81.54

# *These results are based on the set of genes selected by Genis Parra*.

*(Prots represents the number of 248 ultra-conserved CEGs present in genome, % Completeness represents percentage of 248 ultra-conserved CEGs present, Total represents total number of CEGs present including putative orthologs, Average represents the average number of orthologs per CEG and % Ortho represents percentage of detected CEGS that have more than 1 ortholog)*.

### Functional annotation of guar leaf transcriptome

**Figure 2:**
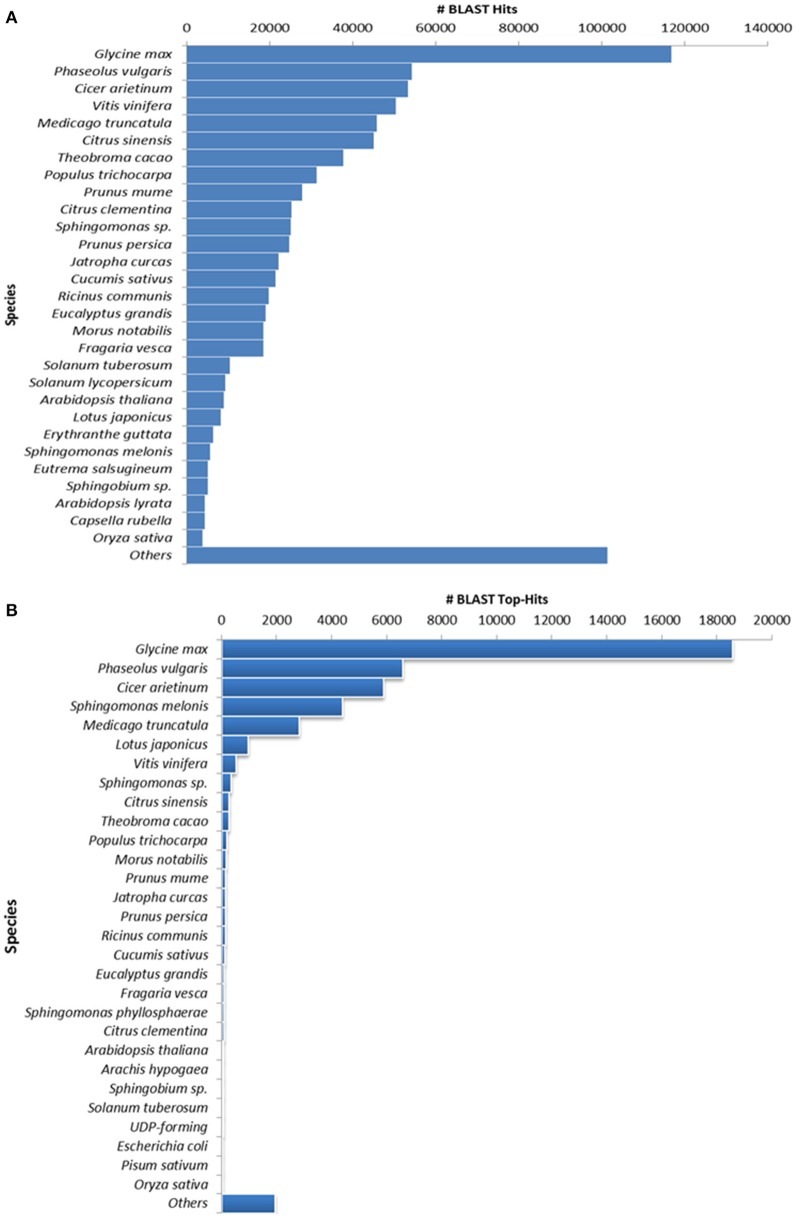
**Species distribution results of guar leaf transcriptome. (A)** By accounting all BLASTX hits and **(B)** Top hit species distribution based on BLASTX alignments.

The annotation of assembled leaf unigenes was done using BLASTX against the Nr, Uniref90, Pfam and Nt databases (Data sheet 1), with an *E*-value cut off of 1e^−6^ (Figure 1B). The total numbers of hits obtained in Uniref90 and Nr databases were 44,992 and 45,972, respectively. Among the 62,146 unigenes, 44,268 (71.23%) had at least one significant match in blast hit results with an *E* < 1e^−6^. Most of these unigenes were found to be protein coding genes. The *E*-value distribution analysis based on Nr database annotation results revealed that 72.29 and 56.65% of the matched sequences had strong homology with the *E*-values < 1e^−30^ and <1e^−45^, respectively. Only 27.70% of the matched sequences showed high similarity with an *E*-value from 1e^−30^ to 1e^−6^ (Figure 1C). The similarity distribution analysis of the BLAST hits indicated that the sequences having a similarity higher than 80% were 66.34% whereas the sequences with a similarity ranging from 35 to 80% were only 33.65% (Figures 1D,E). The species distribution analysis revealed that the sequences homologous to guar unigenes were found in several plant species (Figure 2). The maximum similarity of 41.91% was found with *Glycine max*, followed by *Phaseolus vulgaris* (14.85%), *Cicer arietinum* (13.30%), *Sphingomonas melonis* (9.89%) and *Medicago truncatula* (6.34%). The comparison of assembled unigenes with closely related sequenced species was carried out by TRAPID analysis. Out of total 62,146 assembled unigenes 39,123 (63%), 34,744 (55.9%) and 35,263 (56.7%) showed similarity to *G. max, M. truncatula* and *Lotus japonicus*, respectively. The detailed results of comparison with three species showing the meta annotation, gene family and functional annotation information have been presented in Supplementary Table S2.

**Figure 3:**
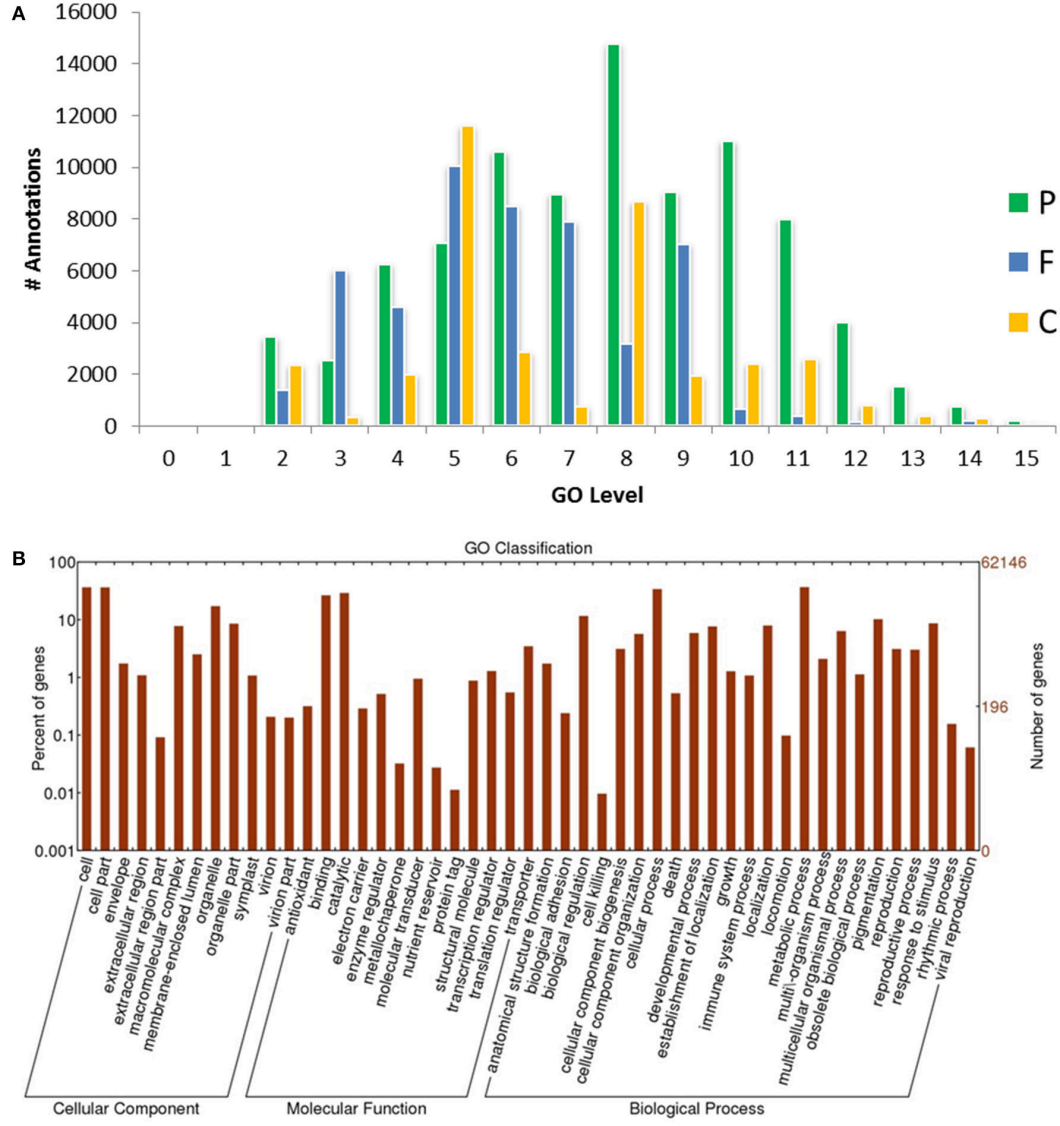
**GO-level distributions in guar leaf transcriptome. (A)** P, F and C represent the biological process, molecular function and cellular component, respectively. Total Annotations = 175,882, Mean Level = 7.011, and **(B)** Classification of guar leaf transcripts into functional categories according to GO-terms on the basis of WEGO tool.

**Figure 4:**
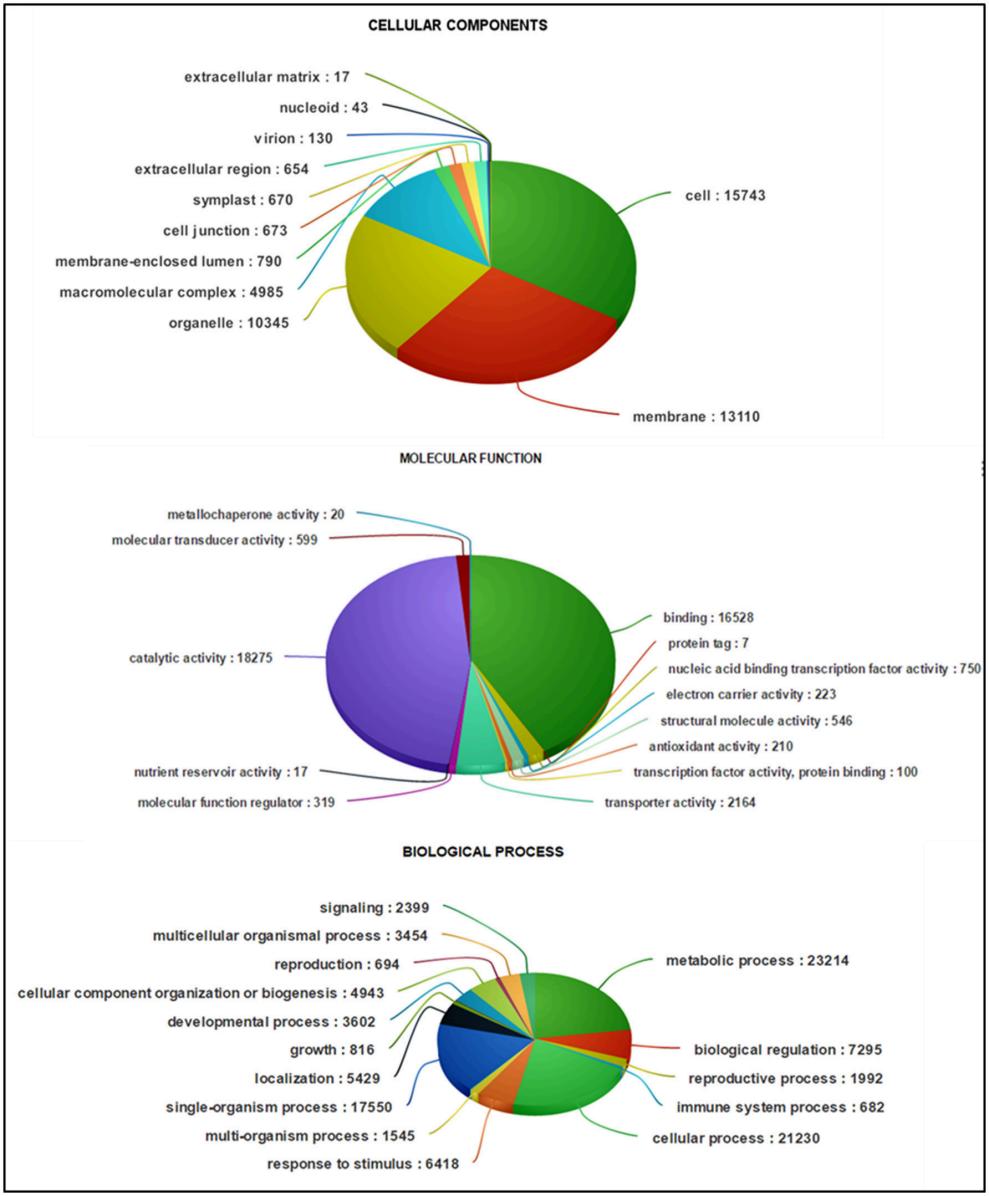
**Classification of guar leaf transcripts into functional categories according to GO-terms**.

Based on sequence homology, 62,146 Trinity-assembled guar leaf unigenes were assigned GO terms. A total of 175,882 annotations were found on the basis of BLAST+ results (Figure 3A). These GO terms were distributed into 46 functional groups, which were further classified into three categories, namely, cellular component, molecular function and biological process (Figure 4). The top GO terms were “metabolic process” (23,214), “cellular process” (21,230), “single-organism process” (17,550) and “biological regulation” (7295) in the biological process category. In the molecular function category, “catalytic activity” (18,275), “binding” (16,528) and “transporter activity” (2164) were major GO terms. In the cellular component category, “cell” (15,743), “membrane” (13,110), “organelle” (10,345) and “macromolecular complex” (4985) were mainly enriched. Only a few unigenes were classified in terms of “cell killing,” “behavior,” “protein tag,” “translation regulator activity,” “nutrient reservoir activity,” and “extracellular matrix.” Similar results were obtained by using WEGO tool (Figure 3B).

By searching against the available database, a total of 11,308 guar unigenes were annotated with various enzyme codes (Data sheet 2). The annotated enzyme codes were grouped into six classes: Oxidoreductases (17.97%), Transferases (41.04%), Hydrolases (26.51%), Lyases (4.61%), Isomerases (3.61%), and Ligases (6.26%) as shown in Figure 1F.

**Figure 5:**
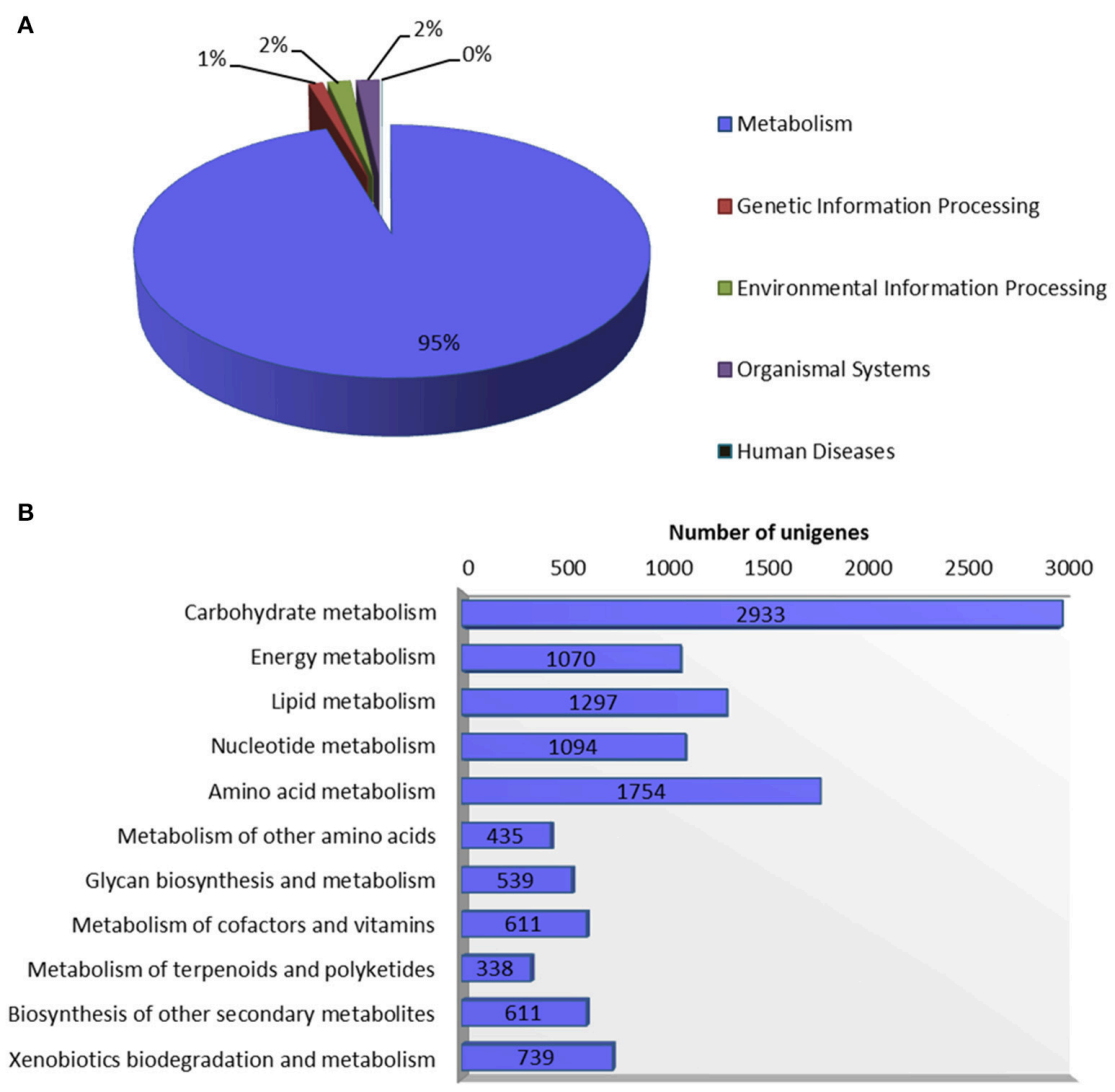
**Annotation of guar leaf transcriptome in KEGG database. (A)** Distribution of unigenes into KEGG biological categories. **(B)** Classification of unigenes in KEGG “metabolism” category.

Systematic high-level gene function analysis against KEGG database resulted in assigning biochemical pathways to 11,971 guar leaf unigenes. These unigenes were associated with 145 KEGG maps and 1759 enzyme codes (Supplementary Table S3). The annotated unigenes were categorized into five major pathways in KEGG database—“metabolism” (11,421), “genetic information processing” (132), “environmental information processing” (207), “organismal systems” (208), and “human diseases” (3). The “metabolism” was the most highly represented category which led to in-depth analysis of this group (Figure 5). The top five enriched pathways were “carbohydrate metabolism” (2933), “amino acid metabolism” (1754), “lipid metabolism” (1297), “nucleotide metabolism” (1094) and “energy metabolism” (1070).

### Identification of differentially expressed genes

Two guar varities, namely, M-83 and RGC-1066 showed ~80% similar gene expression in leaf transcriptome. A total of 175 unigenes were found to be overexpressed with at least 30-folds overexpression in variety M-83. These unigenmes were further annotated against KEGG databse and 36 KEGG maps with 49 ECs were found. A total of 158 unigenes were found in RGC-1066 variety with overexpression of 20-folds and only two KEGG maps with five EC were annotated (Supplementary Table S7).

### Identification of simple sequence repeats (SSRs)

**Figure 6:**
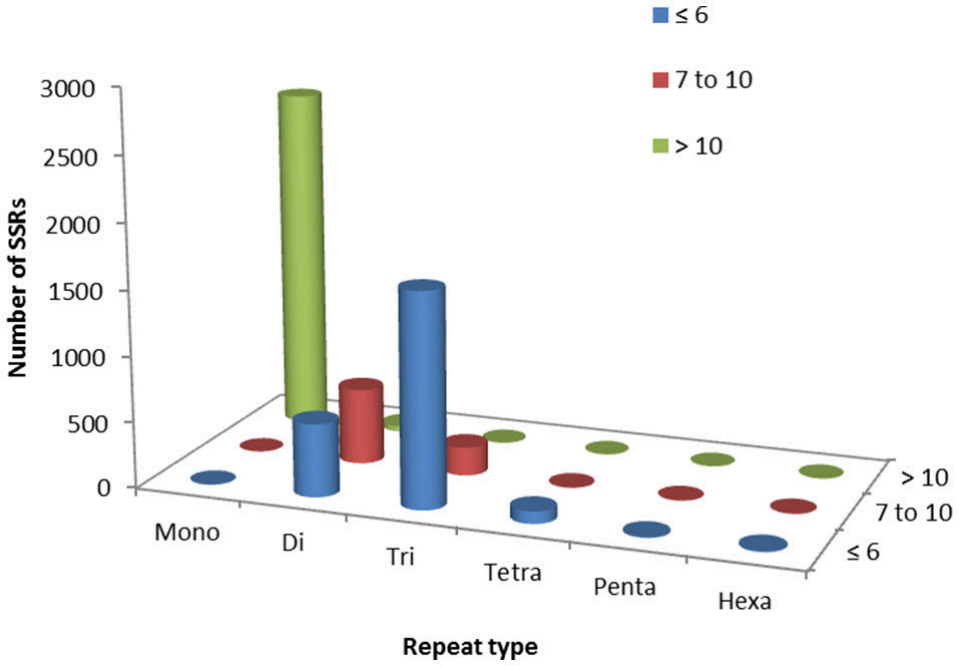
**Simple sequence repeat (SSR) mining results in guar leaf transcriptome**.

Out of total 62,146 unigenes assembled in guar leaf transcriptome, 4970 unigenes were found to contain 5773 SSRs (Data sheets 3, 4). More than one SSR was present in 593 unigenes. On an average basis, one SSR per 7.31 kb was found in the unigenes. The SSRs contained 2624 (45.45%) mononucleotide, 1179 (20.42%) dinucleotide, 1856 (32.14%) trinucleotide, 97 (1.68%) tetranucleotide, 7 (0.12%) pentanucleotide, and 10 (0.17%) hexanucleotide motifs (Figure 6). Most of the SSRs were not repeated more than 10 times. Only a small number of SSRs with more than 20 repeat sequences were observed (Table 3). For most dinucleotide SSRs, the repeat numbers varied from 6 to 11, with 9.92 average value, while the repeat numbers of most of the pentanucleotide and hexanucleotide types were <6. If the mononucleotide SSRs were excluded, trinucleotide repeats were found to be the maximum (1856).

**Table 3:** **Profiles of different SSR types in guar leaf transcriptome**.

**Repeat type**	**Repeat numbers**			**Total**
	**≤ 6**	**7 to 10**	**>10**	
Mononucleotide	0	0	2624	2624
Dinucleotide	559	578	42	1179
Trinucleotide	1636	218	2	1856
Tetranucleotide	93	3	1	97
Pentanucleotide	6	1	0	7
Hexanucleotide	9	1	0	10

**Figure 7:**
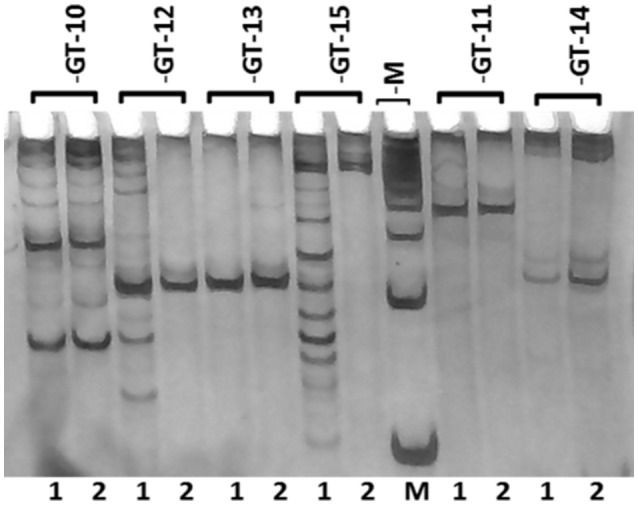
**Banding pattern of SSR primers' amplification on genomic DNA of guar**. M represents 100 bp marker and Lanes 1 and 2 represent guar varieties M-83 and RGC-1066, respectively.

A total of 20 SSR markers representing all the repeat motifs (except mononucleotide repeats) in the *de novo* transcriptome assembly were selected for wet laboratory validation. The flanking primers were designed for SSR containing sequences using the online tool Primer3. Five primers for each dinucleotide, trinucleotide and tetranucleotide repeats, three primers for each pentanucleotide repeat and two primers for each hexanucleotide repeat, were designed and synthesized. The details of the transcriptome sequence ID, motif type and SSR length are given in Supplementary Table S4. The details of the primers synthesized are shown in Supplementary Table S5. Out of the 20 primer pairs, 13 (GT-2, 3, 5, 6, 7, 8, 9, 10, 11, 12, 13, 14, and 18) resulted in PCR amplification in the two guar varieties. Three primer pairs (GT-16, 17, and 19) showed amplification only in the variety RGC-1066 whereas the SSR primer pair GT-15 resulted in amplification only in M-83 variety. The SSR primer GT-17 showed amplification at higher size than the theoretical amplicon size. Some of the tested markers showed more than one band and no polymorphism was detected in the tested SSR primers. The results of amplification of six primer pairs are shown in Figure 7. Figures of PCR amplification results of other primers are not shown. Some of the tested markers showed more than one band that might be due to the presence of multiple sites complementary to the primers in the genomic DNA. Only 65% of the 20 tested SSR primers resulted in amplification in the target guar varieties M-83 and RGC-1066.

### *In silico* identification of SSR polymorphism

The reads of each guar variety were mapped against the assembled unigenes to obtain the sorted transcripts (BAM files). The overall alignment rates were found to be 89.44 and 91.69% for guar varieties M-83 and RGC-1066, respectively. The sorted transcripts were further aligned against the reference by IGV 2.3 software and observed manually to get the nucleotide differences surrounding the SSR region in both varieties. As a result, a total number of 145 SSRs were found to be polymorphic between the two guar varieties (Supplementary Table S6). Two instances of *in silico* polymorphic SSRs have been shown in Supplementary Figure S1.

### Detection of single nucleotide polymorphisms (SNPs)

**Figure 8:**
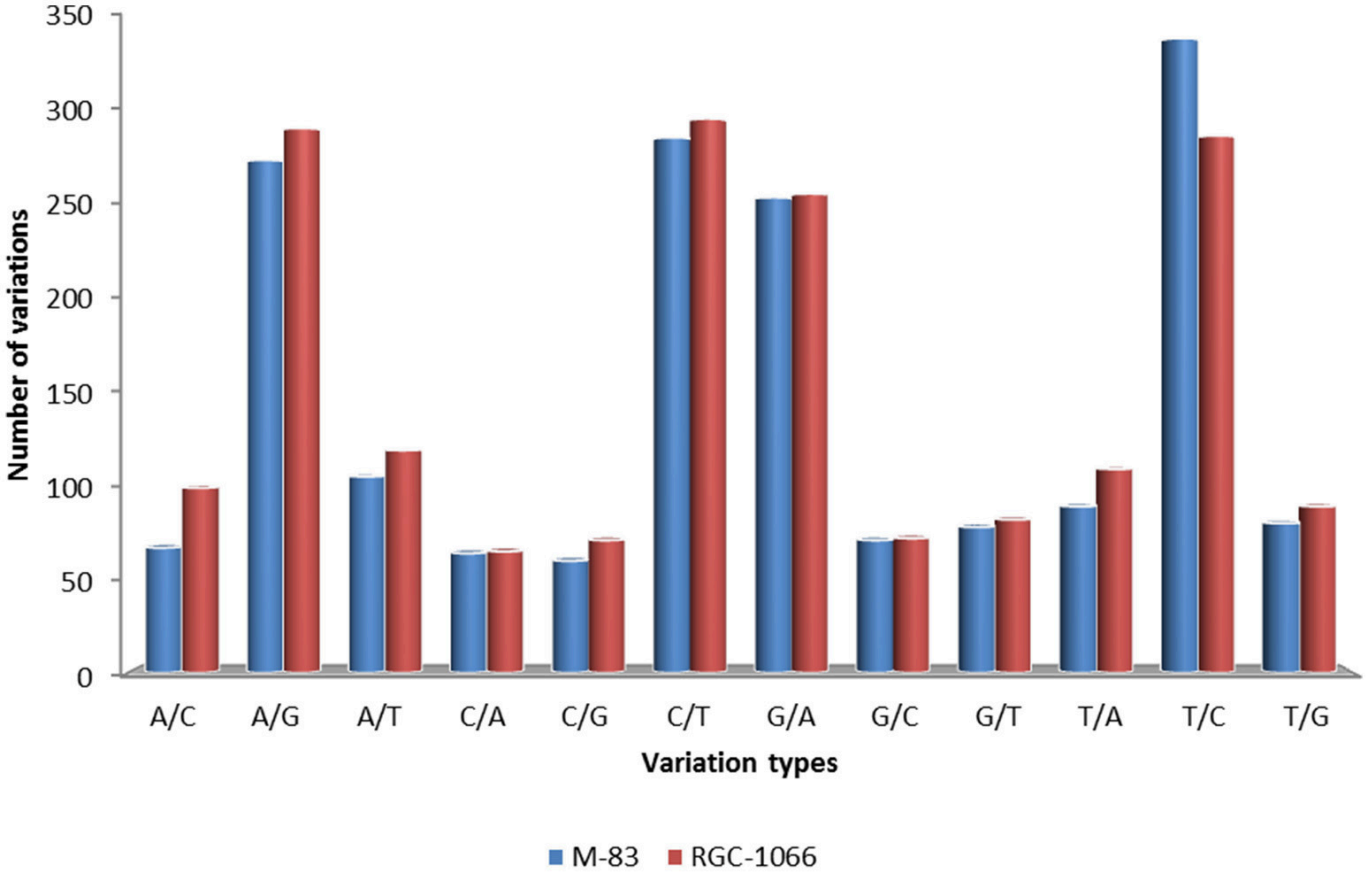
**Statistical information of SNPs in guar varieties M-83 and RGC-1066 against *de novo* assembly**.

A total of 53,402 putative SNPs (~1 SNP per transcript) were identified and out of these 8416 were found with the read depth of >5. These results showed that about one SNP was present for every 5.01 kb of leaf transcriptome in guar. High-confidence 3594 SNPs were obtained after filtering for homozygous SNPs (Data sheet 5). The statistical analysis of SNP loci was done for each variety against the assembled transcripts. This resulted in 65.25% transition nucleotide substitutions and 34.75% transversions in guar variety M-83. In variety RGC-1066 61.36% transitions and 38.64% transversions were found. The statistical information of SNPs in guar varieties M-83 and RGC-1066 against the reference is shown in Figure 8. In addition, 2930 and 3984 Insertion-Deletion (InDel) variants were found in the varieties M-83 and RGC-1066, respectively.

## Discussion

Guar (*Cyamopsis*) is an exclusively diploid (2n = 14) genus with haploid chromosome number 7. The genome sizes (4C DNA contents) in all its three species, viz., *C. tetragonoloba, C. serrate*, and *C. senegalensis*, have been reported to be 10.05, 20.35, and 18.19 pg, respectively (Patil, 2004). The nuclear genomes of legumes vary greatly in size, from 370 million base pairs (Mbp) in *Lablab niger* to more than 13,000 Mbp in the genome of *Vicia faba*. Most of the cultivated species are modest in genome size; mung bean, cowpea, common bean, chick pea, and clover all have haploid genomes smaller than 1000 Mbp (Young et al., 2003). The genome of *Cyamopsis* in comparison to the other legumes, is intermediate in size. Despite the intermediate size of the guar genome, very few studies have been done on the molecular genetics of this crop.

All genetic improvement programs in guar have been carried out till now using conventional breeding without the involvement of molecular markers. As a result, only a limited success has been achieved in obtaining improved guar varieties. Marker assisted breeding, especially with SSRs and SNPs, has given excellent results in several other crops (Rafalski, 2002; Kesawat and Kumar, 2009; Hiremath et al., 2012). Such breeding programs have not been possible in guar due to the lack of sufficient number of SSRs (Kuravadi et al., 2014; Kumar et al., 2016) and the complete absence of SNPs. This has happened due to the limited availability of genetic resources in this crop. NGS technologies provide novel opportunities not only in functional genomics and gene discovery but also in developing huge genetic resources in non-model plants (Wang et al., 2009). These technologies have been widely used for the development of molecular markers through transcriptome analysis in several plant species (Dutta et al., 2011; Wang et al., 2011, 2014).

The present study was performed on two guar varieties, one gum producing variety having hairy leaves (RGC-1066) and the other vegetable variety having pubescent leaves (M-83). Approximately 60 MB high quality sequence reads from the leaf tissues of both guar varieties were assembled to generate 62,146 unigene contigs which represented a large fraction of the guar transcriptome and helped in identification of a comprehensive set of genic-markers. The *de novo* assembly indicated good coverage as well as the depth of sequencing data. The CEGMA software was used for assessment of completeness of a transcriptome assembly by evaluating the presence and completeness of the widely conserved set of 248 CEGs. These CEGs represent the proteins mostly coded by the housekeeping genes and therefore can be expected to be expressed (Parra et al., 2007, 2009; Nakasugi et al., 2013). The CEGMA analysis revealed that assembly had 87.50% of complete and 97.18% partial CEGs. Similar results were obtained in *de novo* transcriptome assembly of *Nicotiana benthamiana* (Nakasugi et al., 2013). Hence the *de novo* assembly obtained in this work was appropriate for the functional annotation and identification of genic markers.

Guar being a non-model plant and without any prior genome information, sequence similarity search and comparison for the assembled unigenes of guar leaf transcriptome were carried out by BLASTX against several databases. The total numbers of hits obtained in Uniref90 and Nr databases were 44,992 and 45,972, respectively. Among the 62,146 unigenes, 71.23% had at least one significant match in blast hit results with an *E* < 1e^−6^ showing that most of the unigenes code for proteins. The unigenes that had no significant matches may be lacking a known conserved functional domain or are representing non-coding RNAs. Another explanation could be that these unigenes, despite containing a known protein domain, do not show sequence matches as they are very short (Wu et al., 2015). Moreover, as very little genomic and transcriptomic information is available for guar, many guar lineage specific genes may not be present in the available databases. The part of sequences showing no hits might be of great interest for further research for alternative splice variants, novel gene products and differentially expressed genes. As per species distribution analysis a number of sequences homologous to guar leaf sequences are present in many plant species. Among these plant species *G. max* genes have the highest similarity (41.91%) with guar unigenes. Hence for the transcriptome analysis of guar, the genome of *G. max* may serve as a reference.

The GO database is an important resource as GO terms provide a set of dynamically controlled and structured vocabularies for describing the roles of genes in any organism (Ashburner et al., 2000). Based on sequence homology, 62,146 guar leaf transcriptome unigenes were assigned GO terms and classified into three main categories, namely, cellular component, molecular function, and biological process. The annotation of guar unigenes with enzyme codes revealed that non-specific serine/threonine protein kinases, phosphoprotein phosphatases, and RNA helicases were most abundant. The above findings are consistent with the other plant leaf transcriptome studies (Wu et al., 2015; Bose Mazumdar and Chattopadhyay, 2016). The identification of several enzyme codes of guar in this work is likely to be helpful in understanding various metabolic activities of this industrially important crop.

The gene function analysis against KEGG database revealed that 11,971 guar leaf unigenes were assigned with 145 KEGG pathways and 1759 enzyme codes. It was observed that more than one unigenes were annotated with the same enzyme in our dataset. Similar pattern was also found in *P. amarus* leaf transcriptome (Bose Mazumdar and Chattopadhyay, 2016). Transcriptome profiling by RNA-Seq has enabled comparison of transcriptional variation in two guar varieties. Both the varieties showed ~80% similar gene expression in leaf transcriptome. The direct comparison of expression of genes would require a meta-analysis (Bhargava et al., 2013) to have a better insight into the functions of genes specifically and commonly involved in various leaf characteristics.

Our main goal in this study was to identify genic-markers that can be readily used in breeding programs. Among various molecular markers, SSRs and SNPs are the most useful ones for genetics and plant breeding applications (Hiremath et al., 2012). In the present study, two sets of molecular markers, SSR and SNP were identified using the transcriptome dataset of guar leaves. Transcriptome based markers are advantageous as compared to the markers in non-transcribed regions due to their high amplification rates and cross-species transferability (Barbará et al., 2007). A total of 5773 potential SSRs were identified with an average of one SSR per 7.31 kb in the unigenes. This result was consistent with the previous EST-SSR report in guar with occurrence (kb/SSR) of 7.9 (Kumar et al., 2016) while, Kuravadi et al. reported the occurrence of 4.1 using the same dataset (Kuravadi et al., 2014). The occurrence of genic-SSR was also comparable to 8.4 in pigeonpea, 3.4 in rice, 5.4 in wheat, and 7.4 in soybean (Cardle et al., 2000; Peng and Lapitan, 2005; Dutta et al., 2011). The differences in genic-SSR abundance may be due to the size of EST or unigene assembly dataset, and different data mining tools and criteria (Varshney et al., 2005a). The frequency distribution of SSR markers are in agreement of previous reports in guar (Kuravadi et al., 2014; Kumar et al., 2016). If the mononucleotide SSRs are excluded because of the frequent homopolymer errors found in sequencing data, a large proportion was covered by di- and trinucleotides (96%) while the rest amounted to <4%. This is consistent with the EST-SSRs distributions reported in many legumes (Wang et al., 2014). A similar trend was observed in other plant species (Sonah et al., 2011; Ahn et al., 2013). The trinucleotide repeats, which are more frequently detected in coding regions, have been reported to be the maximum (Yu et al., 2011). The possible reason for abundance in trinucleotide motifs may be due to expansion or contraction of di-nucleotide repeat length in exons to suppress deleterious effects of the frame-shift mutations in translated regions (Xin et al., 2012). These repeats are generally more robust since they are reported to give fewer “stutter bands” than the dinucleotide repeats. The trinucleotide repeats have been reported as highly polymorphic and stably inherited (Yang et al., 2012). The 5773 potential SSRs identified from *de novo* transcriptome sequencing data of guar leaf represent a significant addition to the limited set of genic-SSR markers available in guar.

The results of SSR markers validation showed that 13 of the 20 tested SSR primers resulted amplification in the target guar varieties M-83 and RGC-1066. The lack of amplification of 7 SSR markers could be because of the flanking primers extending across a splice site with a large intron or chimeric cDNA contigs (Varshney et al., 2006). Some of the tested markers showed more than one band that might be due to the presence of multiple sites complementary to the primers in the genomic DNA. None of the tested markers showed distinct polymorphism. The possible reason may be due to the small product size difference or actual lack of polymorphism as earlier reported in pigeonpea (Dutta et al., 2011). Overall 65% of the tested SSRs were validated successfully by wet laboratory analysis. These results are consistent with barley, where 67–70% of the primers showed amplification (Thiel et al., 2003; Varshney et al., 2006). The amplification success rate was higher than that reported sugarcane (48%) and lower than flax (92%; Cordeiro et al., 2001; Cloutier et al., 2009). *In silico* polymorphism analysis of the SSR markers was done by IGV software (Thorvaldsdóttir et al., 2013). A total of 145 out of 5773 SSR markers were identified as *in silico* polymorphic in the guar varieties M-83 and RGC-1066. This result is in agreement with the reports in pigeonpea (Dutta et al., 2011). Taken together with the previous SSR polymorphism studies, it can be concluded that genetic diversity in the guar gene pool is very low (Kuravadi et al., 2014; Kumar et al., 2016).

A total number of 53,402 putative SNPs (~1 SNP per transcript) were detected in the two guar varieties M-83 and RGC-1066. The putative SNPs were screened for a minimum depth of five reads with same homozygous allele. The screening process might have reduced the sensitivity in detecting rare SNPs, but the probability of true SNP detection was increased due to the reduced chances of inclusion of false variants that arise by sequencing errors. High-confidence differences were composed of 3594 SNPs after screening for the SNP density. SNPs are genetic markers which are bi-allelic in nature, besides being highly abundant and less prone to mutations as compared to SSRs. They can contribute directly to a phenotype or can be associated with a phenotype as a result of linkage disequilibrium (Neff et al., 1998). In plants, SNPs are particularly useful in the construction of high resolution genetic maps, the positional cloning of target loci, marker assisted breeding of important genes, genome wide large-scale linkage disequilibrium associate analysis, DNA fingerprinting, and species origin, relationship and evolutionary studies (Shahinnia and Sayed-Tabatabaei, 2009). Most conventional molecular markers, such as restriction fragment length polymorphism (RFLP) and cleaved amplified polymorphic sequence (CAPS), are based on SNPs, i.e., nucleotide substitutions or insertions/deletions (Nasu et al., 2002). The existence of a restriction site difference spanning the SNPs between varieties/lines to be analyzed is essential for converting SNPs to CAPS markers. However, Michaels and Amasino (1998) and Neff et al. (1998) demonstrated that single-base changes generating no restriction site differences could be employed for the development of PCR-based markers by the derived CAPS (dCAPS) method. Like the CAPS markers, the dCAPS markers are simple and relatively inexpensive to identify (Neff et al., 1998).

Statistical analysis of SNP loci resulted in 65.25% transition nucleotide substitutions and 34.75% transversions in guar variety M-83. In variety RGC-1066 61.36% transitions and 38.64% transversions were found. This finding is in agreement with red pepper transcriptome profiling (Lu et al., 2012). These results of higher occurrence of transitions in comparison to transversions are in accordance of transition/transversion rate bias. Transitions (T↔C and A↔G) have been found to occur at higher frequencies than transversions or all other changes in almost all studied genomes (Gojobori et al., 1982; Wakeley, 1994, 1996; Yang and Bielawski, 2000). The detection of transition/transversion rate bias is important to understand the patterns of DNA sequence evolution and phylogeny reconstruction (Yang and Yoder, 1999).

This study is the first report of transcriptome analysis and SNPs detection in guar crop. The large number of SSRs and SNPs identified in this study provide a wealth of potential markers in this crop. These results are expected to open new opportunities for population genetics, linkage mapping, comparative genomics and marker-assisted breeding in guar.

## Conclusions

The transcriptome sequencing of leaf tissues from two guar varieties, namely, M-83 and RGC-1066 was done by Illumina HiSeq technology. Approximately 30 million pair-end reads of each variety were used to generate a *de novo* assembly of 62,146 unigenes with an average length of 679 bp. The assembled unigenes were functionally annotated against non-redundant protein (Nr), Gene Ontology (GO), and KEGG databases. The genic markers identification resulted in a total of 5773 potential SSRs and 3594 high-quality SNPs. Twenty SSRs were validated using wet laboratory analysis and 145 SSRs were found to be polymorphic by *in silico* polymorphism detection. Taken together, this study not only reports the first transcriptomic dataset and SNPs in guar, but also provides the largest genetic resource in this crop for marker-assisted breeding, functional genomics, and proteomics research in future.

## Author contributions

UT planned the experiments, did experimental work, analyzed the data, made conclusions, and wrote the paper. VP planned the experiments, interpreted the results and gave suggestions on the manuscript. GR planned the experiments, interpreted the results, and corrected the manuscript.

### Conflict of interest statement

The authors declare that the research was conducted in the absence of any commercial or financial relationships that could be construed as a potential conflict of interest.

## Supplementary material

Supplementary Figure S1 **The instances of *in silico* identified polymorphic SSR markers. (A)** comp9618_c0_seq1106-155 and **(B)** comp11342_c0_seq12,182-2,233.
