# Supplementary material for: RNA-Seq of Guar (Cyamopsis tetragonoloba, L. Taub.) Leaves: De novo Transcriptome Assembly, Functional Annotation and Development of Genomic Resources
Source: Front Plant Sci. 2017 Feb 2;8:91. doi: 10.3389/fpls.2017.00091 (PMC5288370; doi:10.3389/fpls.2017.00091)
Supplement: Supplementary file 1 [file Table1.DOCX]

**Supplementary Table S1: Summary of quality assessment report of raw reads of guar leaf transcriptomes**

| **Sl. No.** | **Sample** | **Read Orientation** | **Mean Read Quality (Phred Score)** | **Number of reads** | **% GC** | **% Q<10** | **% Q 10-20** | **% Q 20-30** | **% Q>30** | **Number of Bases (MB)** | **Mean Read Length** |
| --- | --- | --- | --- | --- | --- | --- | --- | --- | --- | --- | --- |
| **1** | M-83 | R1 | 35.36 | 28688024 | 49.81 | 2.2 | 1.12 | 6.01 | 90.67 | 2868.8 | 100.0 |
|  |  | R2 | 34.45 | 28688024 | 49.61 | 4.45 | 1.24 | 6.19 | 88.12 | 2868.8 | 100.0 |
| **2** | RGC-1066 | R1 | 35.92 | 33018878 | 45.86 | 1.52 | 0.91 | 5.04 | 92.54 | 3301.89 | 100.0 |
|  |  | R2 | 35.43 | 33018878 | 45.51 | 2.9 | 0.96 | 4.81 | 91.33 | 3301.89 | 100.0 |
