# Supplementary material for: RNA-Seq of Guar (Cyamopsis tetragonoloba, L. Taub.) Leaves: De novo Transcriptome Assembly, Functional Annotation and Development of Genomic Resources
Source: Front Plant Sci. 2017 Feb 2;8:91. doi: 10.3389/fpls.2017.00091 (PMC5288370; doi:10.3389/fpls.2017.00091)
Supplement: Supplementary file 2 [file Table2.DOCX]

**Supplementary Table S2: Comparison of guar assembled leaf transcriptome with closely related sequenced species using TRAPID Analysis**

| **Transcript Information** | | | | | | |
| --- | --- | --- | --- | --- | --- | --- |
| Unitranscripts | | | | 62,146 | | |
| Average unitranscript length | | | | 679.4 bp | | |
|  | | ***Glycine max*** | | | ***Medicago truncatula*** | ***Lotus japonicus*** |
| **Meta Annotation Information** | | | | | | |
| Meta annotation full-length | | **7999 (12.9%)** | | | 7259 (11.7%) | 6239 (10%) |
| Meta annotation quasi full-length | | 14948 (24.1%) | | | 13472 (21.7%) | 11058 (17.8%) |
| Meta annotation partial | | 15764 (25.4%) | | | 13839 (22.3%) | 11411 (18.4%) |
| Meta annotation no information | | 23435 (37.7%) | | | 27576 (44.4%) | 33438 (53.8%) |
| **Similarity Search Information** | | | | | | |
| Similarity | 39123 (100%) | | | | 34744 (100%) | 35263 (100%) |
| **Gene Family Information** | | | | | | |
| Gene families | | | 6454 | | 5307 | 8382 |
| Unitranscripts in GF | | | 39123 (63%) | | 34744 (55.9%) | 35263 (56.7%) |
| **Functional Annotation Information** | | | | | | |
| Unitranscripts with GO | | | 29981 (48.2%) | | 26607 (42.8%) | 24079 (38.7%) |
| Unitranscripts with Protein Domain | | | 34407 (55.4%) | | 30593 (49.2%) | 28611 (46%) |
