# Supplementary material for: RNA-Seq of Guar (Cyamopsis tetragonoloba, L. Taub.) Leaves: De novo Transcriptome Assembly, Functional Annotation and Development of Genomic Resources
Source: Front Plant Sci. 2017 Feb 2;8:91. doi: 10.3389/fpls.2017.00091 (PMC5288370; doi:10.3389/fpls.2017.00091)
Supplement: Supplementary file 4 [file Table4.DOCX]

**Supplementary Table S4: Details of SSR markers used for validation in guar varieties M-83 and RGC-1066**

| **ID** | **SSR No.** | **SSR Type** | **SSR** | **Size** | **Start** | **End** |
| --- | --- | --- | --- | --- | --- | --- |
| **Dinucleotide** |  |  |  |  |  |  |
| comp5733_c0_seq1_len=241 | 1 | p2 | (GA)6 | 12 | 92 | 103 |
| comp15714_c1_seq1_len=293 | 1 | p2 | (CT)8 | 16 | 206 | 221 |
| comp23394_c0_seq1_len=298 | 1 | p2 | (CT)6 | 12 | 155 | 166 |
| comp23819_c0_seq1_len=301 | 1 | p2 | (TC)6 | 12 | 175 | 186 |
| comp1834_c0_seq1_len=232 | 1 | p2 | (AG)9 | 18 | 122 | 139 |
| **Trinuceotide** | | | | | | |
| comp19070_c0_seq1_len=655 | 1 | p3 | (GCC)5 | 15 | 407 | 421 |
| comp23428_c0_seq1_len=1506 | 1 | p3 | (TGG)7 | 21 | 1386 | 1406 |
| comp18850_c0_seq1_len=1876 | 1 | p3 | (TTC)6 | 18 | 434 | 451 |
| comp17598_c0_seq1_len=432 | 1 | p3 | (TCT)5 | 15 | 197 | 211 |
| comp19082_c0_seq1_len=464 | 1 | p3 | (CAT)5 | 15 | 302 | 316 |
| **Tetranucleotide** | | | | | | |
| comp23741_c0_seq1_len=2956 | 1 | p4 | (CTTT)5 | 20 | 236 | 255 |
| comp50026_c0_seq1_len=304 | 1 | p4 | (ATAG)5 | 20 | 187 | 206 |
| comp48573_c0_seq1_len=1530 | 1 | p4 | (TCAC)6 | 24 | 181 | 204 |
| comp274511_c0_seq1_len=339 | 1 | p4 | (TGGT)9 | 36 | 222 | 257 |
| comp33177_c0_seq1_len=5191 | 4 | p4 | (TATC)5 | 20 | 2934 | 2953 |
| **Pentanucleotide** | | | | | | |
| comp18299_c0_seq1_len=983 | 1 | p5 | (TTCTT)5 | 25 | 842 | 866 |
| comp29744_c0_seq1_len=2424 | 1 | p5 | (AGAGA)6 | 30 | 2323 | 2352 |
| comp33888_c0_seq1_len=582 | 2 | p5 | (TCTCA)5 | 25 | 472 | 496 |
| **Hexanucleotide** | | | | | | |
| comp148748_c0_seq1_len=369 | 1 | p6 | (AATTCA)5 | 30 | 60 | 89 |
| comp27051_c0_seq1_len=1109 | 1 | p6 | (TGGAGC)5 | 30 | 696 | 725 |
