# Supplementary material for: RNA-Seq of Guar (Cyamopsis tetragonoloba, L. Taub.) Leaves: De novo Transcriptome Assembly, Functional Annotation and Development of Genomic Resources
Source: Front Plant Sci. 2017 Feb 2;8:91. doi: 10.3389/fpls.2017.00091 (PMC5288370; doi:10.3389/fpls.2017.00091)
Supplement: Supplementary file 5 [file Table5.DOCX]

**Supplementary Table S5: Details of primer pairs designed and synthesized for validation of SSR markers in guar varieties M-83 and RGC-1066**

| Sr. No. | **ID** | | **NAME** | **OLIGO** | **Start** | **Length** | **Tm** | **GC%** | **Sequence** | **Product Size** |
| --- | --- | --- | --- | --- | --- | --- | --- | --- | --- | --- |
| **Dinucleotide** | | | | | | | | | | |
| 1 | comp5733 | **GT-01** | | LEFT PRIMER | 32 | 22 | 57.68 | 40.91 | ACTTCATGGTGATGGATTTGGA | 210 |
|  |  |  |  | RIGHT PRIMER | 241 | 23 | 57.63 | 43.48 | CGCTCTTCCGATCTAGAATTAGT |  |
| 2 | comp15714 | **GT-02** | | LEFT PRIMER | 51 | 20 | 60.18 | 55 | GGCAAGGAAAAGGGGTGAGT | 229 |
|  |  |  |  | RIGHT PRIMER | 279 | 20 | 59.58 | 55 | AGAGAAGGAAGGAGGGAGCA |  |
| 3 | comp23394 | **GT-03** | | LEFT PRIMER | 30 | 20 | 58.7 | 50 | ACTGGGATCTGAATTGGGCT | 231 |
|  |  |  |  | RIGHT PRIMER | 260 | 20 | 60.18 | 60 | TCCTCTGCTAGCCTAGTCCG |  |
| 4 | comp23819 | **GT-04** | | LEFT PRIMER | 38 | 22 | 59.49 | 50 | TCCACAGCCTCTTTCTTATCCC | 200 |
|  |  |  |  | RIGHT PRIMER | 237 | 20 | 58.22 | 60 | GAGAGGGACAGGGAGAAGAG |  |
| 5 | comp1834 | **GT-05** | | LEFT PRIMER | 3 | 23 | 58.98 | 43.48 | ACGCTTAGATTAGTGGGTTCTCT | 202 |
|  |  |  |  | RIGHT PRIMER | 204 | 20 | 59.4 | 50 | GGTTGCGTGCATTTTCCTCT |  |
| **Trinucleotide** | | | | | | | | | | |
| 6 | comp19082 | | **GT-06** | LEFT PRIMER | 183 | 21 | 59.2 | 47.62 | TGACTTTGTGAATGCTACCGC | 214 |
|  |  |  |  | RIGHT PRIMER | 396 | 20 | 60.11 | 55 | TGATGCTCTCAATGCTGGGG |  |
| 7 | comp19070 | | **GT-07** | LEFT PRIMER | 244 | 20 | 59.68 | 60 | GGATGGATCGGAGGAAGACG | 236 |
|  |  |  |  | RIGHT PRIMER | 479 | 20 | 59.88 | 55 | TTACCCCTACCCAGTGAGCA |  |
| 8 | comp23428 | | **GT-08** | LEFT PRIMER | 1249 | 20 | 59.75 | 60 | GGGAGCTGAAGACAAGAGGG | 200 |
|  |  |  |  | RIGHT PRIMER | 1448 | 20 | 58.95 | 50 | TCGACCAACAATGTCCCAGA |  |
| 9 | comp18850 | | **GT-09** | LEFT PRIMER | 205 | 21 | 59.38 | 47.62 | TGCGATCTGGGAGTTTCAAGA | 454 |
|  |  |  |  | RIGHT PRIMER | 658 | 20 | 59.97 | 55 | CTTGCCCACCTTGAAACTGC |  |
| 10 | comp17598 | | **GT-10** | LEFT PRIMER | 82 | 20 | 60.04 | 55 | CTTCTCCGCGGTTTCTTCCT | 310 |
|  |  |  |  | RIGHT PRIMER | 391 | 20 | 59.62 | 50 | GTCAACAGGTGCGTCGTTTT |  |
| **Tetranucleotide** | | | | | | | | | | |
| 11 | comp23741 | | **GT-11** | LEFT PRIMER | 129 | 20 | 60.04 | 60 | GGACACCGGAGTAAACAGGG | 380 |
|  |  |  |  | RIGHT PRIMER | 508 | 20 | 59.82 | 60 | GGCTTATCCTCCCACCCTTG |  |
| 12 | comp50026 | | **GT-12** | LEFT PRIMER | 61 | 20 | 59.9 | 55 | GATGCCCAATGATGCACCAC | 229 |
|  |  |  |  | RIGHT PRIMER | 289 | 25 | 59.71 | 40 | TCATAGCTTAGAACAAATCACGCAG |  |
| 13 | comp48573 | | **GT-13** | LEFT PRIMER | 157 | 20 | 60.32 | 60 | CCAGCCACCACACTCTTCTC | 234 |
|  |  |  |  | RIGHT PRIMER | 390 | 20 | 60.03 | 55 | AAGGGCAGCTCTAGAGACGA |  |
| 14 | comp274511 | | **GT-14** | LEFT PRIMER | 60 | 20 | 59.46 | 60 | GTCCTCTGTCTTGGCTACCC | 232 |
|  |  |  |  | RIGHT PRIMER | 291 | 20 | 60.32 | 60 | CTCCTTTACCACCTTGCCCC |  |
| 15 | comp33177 | | **GT-15** | LEFT PRIMER | 2568 | 20 | 60.03 | 60 | TGGGATGGTGAGAGGAGAGG | 451 |
|  |  |  |  | RIGHT PRIMER | 3018 | 20 | 60.04 | 50 | ATACGGCGGTGTTGGACATT |  |
| **Pentanucleotide** | | | | | | | | | | |
| 16 | comp18299 | | **GT-16** | LEFT PRIMER | 590 | 20 | 60.11 | 55 | CCCCTGCACGAATTGGATCT | 314 |
|  |  |  |  | RIGHT PRIMER | 903 | 20 | 59.97 | 55 | GGAACGGCAACACACTGAAC |  |
| 17 | comp29744 | | **G-17** | LEFT PRIMER | 2113 | 20 | 59.97 | 50 | AAATGGAAGCGTGGTTTGGC | 261 |
|  |  |  |  | RIGHT PRIMER | 2373 | 20 | 58.48 | 60 | TCCTCTCCTCTCCTCTCCTC |  |
| 18 | comp33888 | | **GT-18** | LEFT PRIMER | 139 | 20 | 59.82 | 60 | CTCATGTCCCCTGAACTCGG | 443 |
|  |  |  |  | RIGHT PRIMER | 581 | 20 | 60.86 | 60 | CACGACGCTCTTCCGATCTG |  |
| **Hexanucleotide** | | | | | | | | | | |
| 19 | comp148748 | | **GT-19** | LEFT PRIMER | 40 | 20 | 60.45 | 55 | CTTCTCTTTGCGTCGCGTTG | 255 |
|  |  |  |  | RIGHT PRIMER | 294 | 20 | 60.04 | 55 | ACGACGTTCCCTCCATCAAC |  |
| 20 | comp27051 | | **GT-20** | LEFT PRIMER | 566 | 20 | 59.96 | 55 | TGCCACCATTGTCAGGTCTC | 377 |
|  |  |  |  | RIGHT PRIMER | 942 | 20 | 60.18 | 55 | TGGTCCTCTTCCTCAGCCTT |  |
