# Supplementary material for: RNA-Seq of Guar (Cyamopsis tetragonoloba, L. Taub.) Leaves: De novo Transcriptome Assembly, Functional Annotation and Development of Genomic Resources
Source: Front Plant Sci. 2017 Feb 2;8:91. doi: 10.3389/fpls.2017.00091 (PMC5288370; doi:10.3389/fpls.2017.00091)
Supplement: Supplementary file 6 [file Table6.DOCX]

**Supplementary Table S6: Detailed list of *in silico* identified polymorphic SSR markers in guar leaf transcriptome.**

| **ID** | **SSR type** | **SSR** | **Size** | **Start** | **End** |
| --- | --- | --- | --- | --- | --- |
| comp8464_c0_seq1 | p3 | (TTC)5 | 15 | 314 | 328 |
| comp9618_c0_seq1 | p1 | (T)10 | 10 | 126 | 135 |
| comp11342_c0_seq1 | p2 | (GA)6 | 12 | 2202 | 2213 |
| comp12865_c0_seq1 | p2 | (GA)6 | 12 | 2202 | 2213 |
| comp13809_c0_seq1 | p2 | (AG)9 | 18 | 1569 | 1586 |
| comp13950_c0_seq1 | p1 | (T)12 | 12 | 124 | 135 |
| comp15218_c0_seq1 | p1 | (T)16 | 16 | 97 | 112 |
| comp16454_c0_seq2 | p1 | (A)13 | 13 | 359 | 371 |
| comp16540_c0_seq1 | p1 | (T)12 | 12 | 85 | 96 |
| comp16997_c0_seq1 | p1 | (A)10 | 10 | 105 | 114 |
| comp17604_c0_seq1 | p1 | (A)10 | 10 | 97 | 106 |
| comp17651_c0_seq1 | p1 | (A)11 | 11 | 1990 | 2000 |
| comp17819_c0_seq1 | p3 | (GGA)5 | 15 | 655 | 669 |
| comp17919_c0_seq1 | p1 | (A)10 | 10 | 54 | 63 |
| comp18034_c0_seq1 | p2 | (GA)6 | 12 | 39 | 50 |
| comp18111_c0_seq2 | p2 | (AG)6 | 12 | 8 | 19 |
| comp19155_c0_seq1 | p2 | (AT)7 | 14 | 60 | 73 |
| comp19636_c0_seq2 | p1 | (A)10 | 10 | 96 | 105 |
| comp19724_c0_seq1 | p2 | (AG)6 | 12 | 148 | 159 |
| comp20242_c0_seq1 | p1 | (T)14 | 14 | 222 | 235 |
| comp20346_c0_seq1 | c | (A)10ttattggatgccacatgtcaacatctcattgagtggcgtctgctaggaggcgccattcaatgaggtgctaccacgtgacagcaccagatttttc(A)11 | 115 | 69 | 183 |
| comp20690_c0_seq1 | p1 | (T)13 | 13 | 485 | 497 |
| comp21982_c0_seq1 | p1 | (A)10 | 10 | 142 | 151 |
| comp22227_c0_seq1 | p1 | (A)10 | 10 | 1480 | 1489 |
| comp22347_c0_seq1 | p3 | (CAC)5 | 15 | 707 | 721 |
| comp22851_c0_seq1 | p1 | (T)13 | 13 | 47 | 59 |
| comp22851_c0_seq1 | p1 | (T)13 | 13 | 47 | 59 |
| comp23073_c0_seq1 | p3 | (GGA)5 | 15 | 437 | 451 |
| comp23183_c0_seq1 | p1 | (A)13 | 13 | 73 | 85 |
| comp23487_c0_seq1 | p1 | (A)10 | 10 | 627 | 636 |
| comp24716_c0_seq1 | p3 | (TTC)5 | 15 | 56 | 70 |
| comp24793_c0_seq2 | p1 | (A)10 | 10 | 189 | 198 |
| comp25073_c0_seq1 | p1 | (T)10 | 10 | 708 | 717 |
| comp25073_c0_seq2 | p1 | (T)10 | 10 | 630 | 639 |
| comp25090_c0_seq1 | p2 | (GA)7 | 14 | 2611 | 2624 |
| comp25313_c0_seq1 | p1 | (A)13 | 13 | 147 | 159 |
| comp25594_c0_seq1 | p1 | (A)12 | 12 | 1186 | 1197 |
| comp26054_c0_seq1 | p1 | (T)10 | 10 | 479 | 488 |
| comp26054_c0_seq1 | p1 | (T)10 | 10 | 479 | 488 |
| comp27018_c0_seq1 | p1 | (A)11 | 11 | 1758 | 1768 |
| comp27195_c0_seq1 | p3 | (TCA)5 | 15 | 172 | 186 |
| comp27233_c0_seq1 | p1 | (T)16 | 16 | 106 | 121 |
| comp27233_c0_seq2 | p1 | (T)16 | 16 | 106 | 121 |
| comp27569_c0_seq1 | p1 | (T)11 | 11 | 930 | 940 |
| comp27670_c0_seq2 | c | (GT)6ttggccgat(A)11 | 32 | 167 | 198 |
| comp28095_c0_seq1 | p1 | (A)13 | 13 | 79 | 91 |
| comp28123_c0_seq2 | p1 | (A)13 | 13 | 46 | 58 |
| comp28364_c0_seq1 | p1 | (T)13 | 13 | 34 | 46 |
| comp28512_c0_seq1 | p2 | (AG)8 | 16 | 317 | 332 |
| comp29718_c0_seq4 | p1 | (A)12 | 12 | 927 | 938 |
| comp29859_c0_seq2 | p1 | (A)10 | 10 | 65 | 74 |
| comp29959_c0_seq2 | p3 | (TCA)6 | 18 | 103 | 120 |
| comp29959_c0_seq3 | p3 | (TCA)6 | 18 | 103 | 120 |
| comp30167_c0_seq1 | p1 | (A)11 | 11 | 1285 | 1295 |
| comp30202_c0_seq2 | p1 | (A)13 | 13 | 47 | 59 |
| comp30203_c0_seq1 | p2 | (AG)6 | 12 | 48 | 59 |
| comp30204_c0_seq2 | p1 | (T)10 | 10 | 55 | 64 |
| comp30307_c0_seq1 | p1 | (A)10 | 10 | 37 | 46 |
| comp30576_c0_seq1 | p1 | (A)11 | 11 | 193 | 203 |
| comp30770_c0_seq1 | p2 | (CT)6 | 12 | 169 | 180 |
| comp30801_c0_seq3 | p1 | (T)10 | 10 | 2349 | 2358 |
| comp31001_c0_seq2 | p1 | (T)11 | 11 | 174 | 184 |
| comp31381_c0_seq4 | p1 | (A)16 | 16 | 182 | 197 |
| comp31433_c0_seq1 | p3 | (GAA)6 | 18 | 3364 | 3381 |
| comp31433_c0_seq2 | p3 | (GAA)6 | 18 | 3269 | 3286 |
| comp31433_c0_seq3 | p3 | (GAA)6 | 18 | 3193 | 3210 |
| comp31433_c0_seq4 | p3 | (GAA)6 | 18 | 3098 | 3115 |
| comp32419_c0_seq1 | p2 | (AT)8 | 16 | 2278 | 2293 |
| comp32514_c0_seq2 | p1 | (A)11 | 11 | 91 | 101 |
| comp32762_c0_seq3 | p1 | (A)10 | 10 | 627 | 636 |
| comp32802_c0_seq1 | p1 | (T)15 | 15 | 178 | 192 |
| comp32846_c0_seq1 | p1 | (T)10 | 10 | 349 | 358 |
| comp32856_c0_seq7 | p1 | (T)10 | 10 | 371 | 380 |
| comp33025_c0_seq2 | p3 | (GAT)5 | 15 | 2124 | 2138 |
| comp33036_c0_seq2 | p1 | (A)12 | 12 | 3337 | 3348 |
| comp33091_c0_seq1 | p1 | (A)12 | 12 | 556 | 567 |
| comp33120_c0_seq1 | p1 | (A)14 | 14 | 59 | 72 |
| comp33127_c0_seq1 | p1 | (A)13 | 13 | 270 | 282 |
| comp33127_c0_seq1 | p1 | (A)13 | 13 | 270 | 282 |
| comp33139_c1_seq2 | p1 | (A)12 | 12 | 190 | 201 |
| comp33145_c0_seq13 | p1 | (A)10 | 10 | 196 | 205 |
| comp33193_c0_seq5 | p2 | (AT)9 | 18 | 101 | 118 |
| comp33352_c0_seq1 | p1 | (T)14 | 14 | 109 | 122 |
| comp33366_c0_seq1 | p1 | (A)10 | 10 | 461 | 470 |
| comp33386_c0_seq1 | p3 | (GTT)5 | 15 | 87 | 101 |
| comp33415_c0_seq18 | p3 | (TGG)5 | 15 | 256 | 270 |
| comp33416_c0_seq1 | c | (C)13(A)11 | 24 | 49 | 72 |
| comp33419_c0_seq1 | p1 | (A)10 | 10 | 1664 | 1673 |
| comp33419_c0_seq12 | p1 | (T)10 | 10 | 139 | 148 |
| comp33450_c0_seq7 | c | (A)14caaccaacgcg(CT)6 | 37 | 81 | 117 |
| comp33451_c0_seq1 | p1 | (T)10 | 10 | 1119 | 1128 |
| comp33482_c0_seq13 | p1 | (T)12 | 12 | 547 | 558 |
| comp33514_c0_seq11 | p1 | (A)11 | 11 | 1759 | 1769 |
| comp33521_c0_seq13 | p2 | (AT)6 | 12 | 2693 | 2704 |
| comp33523_c0_seq2 | p3 | (CTG)5 | 15 | 995 | 1009 |
| comp33545_c0_seq3 | p1 | (T)10 | 10 | 151 | 160 |
| comp33601_c0_seq26 | p1 | (A)13 | 13 | 237 | 249 |
| comp33615_c0_seq6 | p1 | (T)11 | 11 | 315 | 325 |
| comp33637_c0_seq4 | p3 | (ATG)5 | 15 | 214 | 228 |
| comp34411_c0_seq1 | p1 | (T)10 | 10 | 114 | 123 |
| comp35672_c0_seq1 | p1 | (T)11 | 11 | 123 | 133 |
| comp37705_c0_seq1 | p1 | (A)17 | 17 | 73 | 89 |
| comp37830_c0_seq1 | c | (A)15gagaatgcaattgtaatcgtcttcgcctgaaaaaacatgcgtgagtttct(TC)6 | 77 | 1772 | 1848 |
| comp38408_c0_seq1 | p1 | (A)10 | 10 | 79 | 88 |
| comp39203_c0_seq1 | p1 | (A)12 | 12 | 136 | 147 |
| comp39524_c0_seq1 | p1 | (T)12 | 12 | 739 | 750 |
| comp40342_c0_seq1 | p4 | (TCTT)6 | 24 | 1617 | 1640 |
| comp50788_c0_seq1 | p1 | (T)11 | 11 | 467 | 477 |
| comp65519_c0_seq1 | p1 | (A)14 | 14 | 417 | 430 |
| comp72045_c0_seq1 | p3 | (AAG)6 | 18 | 439 | 456 |
| comp79376_c0_seq1 | p1 | (T)10 | 10 | 411 | 420 |
| comp86705_c0_seq1 | p1 | (T)10 | 10 | 116 | 125 |
| comp87801_c0_seq1 | p2 | (TC)10 | 20 | 146 | 165 |
| comp89834_c0_seq1 | p1 | (A)14 | 14 | 287 | 300 |
| comp91887_c0_seq1 | c | (A)11caaaaatggaagccagtggtacccatggcatgattttttgttcattccaagttcgcagtt(TG)8 | 87 | 820 | 906 |
| comp95348_c0_seq1 | p1 | (T)10 | 10 | 116 | 125 |
| comp106042_c0_seq1 | p1 | (A)10 | 10 | 48 | 57 |
| comp111064_c0_seq1 | p1 | (A)11 | 11 | 235 | 245 |
| comp121224_c0_seq1 | c | (A)18cgtacgcaactttttg(T)13 | 47 | 188 | 234 |
| comp121500_c0_seq1 | p1 | (T)11 | 11 | 273 | 283 |
| comp122195_c0_seq1 | p3 | (TCT)6 | 18 | 228 | 245 |
| comp128817_c0_seq1 | p1 | (T)12 | 12 | 313 | 324 |
| comp135740_c0_seq1 | p1 | (A)10 | 10 | 63 | 72 |
| comp146132_c0_seq1 | p3 | (GAG)5 | 15 | 238 | 252 |
| comp150496_c0_seq1 | p1 | (T)11 | 11 | 325 | 335 |
| comp184033_c0_seq1 | p1 | (T)12 | 12 | 302 | 313 |
| comp185516_c0_seq1 | p2 | (GA)10 | 20 | 130 | 149 |
| comp187787_c0_seq1 | p1 | (T)13 | 13 | 309 | 321 |
| comp195314_c0_seq1 | p3 | (GTG)5 | 15 | 118 | 132 |
| comp195698_c0_seq1 | p1 | (T)10 | 10 | 81 | 90 |
| comp196550_c0_seq1 | p2 | (GT)6 | 12 | 135 | 146 |
| comp200502_c0_seq1 | p1 | (T)11 | 11 | 82 | 92 |
| comp206092_c0_seq1 | p1 | (T)11 | 11 | 163 | 173 |
| comp217974_c0_seq1 | p1 | (A)10 | 10 | 75 | 84 |
| comp218195_c0_seq1 | p3 | (GAA)6 | 18 | 135 | 152 |
| comp276179_c0_seq1 | p1 | (T)10 | 10 | 61 | 70 |
| comp279255_c0_seq1 | p3 | (ATC)5 | 15 | 248 | 262 |
| comp283457_c0_seq1 | p1 | (T)11 | 11 | 127 | 137 |
| comp289131_c0_seq1 | p1 | (T)10 | 10 | 120 | 129 |
| comp312043_c0_seq1 | p1 | (T)10 | 10 | 97 | 106 |
| comp320230_c0_seq1 | p1 | (T)10 | 10 | 284 | 293 |
| comp362625_c0_seq1 | p1 | (A)10 | 10 | 170 | 179 |
| comp390231_c0_seq1 | p1 | (T)12 | 12 | 113 | 124 |
| comp546252_c0_seq1 | p1 | (A)11 | 11 | 79 | 89 |
| comp556501_c0_seq1 | p1 | (T)11 | 11 | 224 | 234 |
