# Supplementary material for: RNA-Seq of Guar (Cyamopsis tetragonoloba, L. Taub.) Leaves: De novo Transcriptome Assembly, Functional Annotation and Development of Genomic Resources
Source: Front Plant Sci. 2017 Feb 2;8:91. doi: 10.3389/fpls.2017.00091 (PMC5288370; doi:10.3389/fpls.2017.00091)
Supplement: Supplementary file 7 [file Table7.DOC]

**Supplementary Table S7: KEGG pathways showing differentially expressed genes** in guar varieties M-83 and RGC-1066

| **Pathway** | **Pathway ID** | **Guar variety** |
| --- | --- | --- |
| Aminobenzoate degradation | map00627 | M-83 |
| Steroid hormone biosynthesis | map00140 | -do- |
| Glycolysis / Gluconeogenesis | map00010 | -do- |
| Butanoate metabolism | map00650 | -do- |
| Arginine and proline metabolism | map00330 | -do- |
| Chlorocyclohexane and chlorobenzene degradation | map00361 | -do- |
| Pentose and glucuronateinterconversions | map00040 | -do- |
| Methane metabolism | map00680 | -do- |
| Fatty acid degradation | map00071 | -do- |
| Caffeine metabolism | map00232 | -do- |
| Purine metabolism | map00230 | -do- |
| Glycine, serine and threonine metabolism | map00260 | -do- |
| Valine, leucine and isoleucine biosynthesis | map00290 | -do- |
| Carbon fixation in photosynthetic organisms | map00710 | -do- |
| Pantothenate and CoA biosynthesis | map00770 | -do- |
| Limonene and pinene degradation | map00903 | -do- |
| Porphyrin and chlorophyll metabolism | map00860 | -do- |
| Arginine biosynthesis | map00220 | -do- |
| Alanine, aspartate and glutamate metabolism | map00250 | -do- |
| Valine, leucine and isoleucine degradation | map00280 | -do- |
| Thiamine metabolism | map00730 | -do- |
| beta-Alanine metabolism | map00410 | -do- |
| Drug metabolism - other enzymes | map00983 | -do- |
| Glyoxylate and dicarboxylate metabolism | map00630 | -do- |
| Lysine degradation | map00310 | -do- |
| Starch and sucrose metabolism | map00500 | -do- |
| Glycerolipid metabolism | map00561 | -do- |
| Linoleic acid metabolism | map00591 | -do- |
| Arachidonic acid metabolism | map00590 | -do- |
| Vitamin B6 metabolism | map00750 | -do- |
| Starch and sucrose metabolism | map00500 | -do- |
| Glycerolipid metabolism | map00561 | -do- |
| Linoleic acid metabolism | map00591 | -do- |
| Arachidonic acid metabolism | map00590 | -do- |
| Carbon fixation pathways in prokaryotes | map00720 | -do- |
| Vitamin B6 metabolism | map00750 | -do- |
| Purine metabolism | map00230 | RGC-1066 |
| Thiamine metabolism | map00730 | -do- |
