# Supplementary figures and images for: RNA-Seq of Guar (Cyamopsis tetragonoloba, L. Taub.) Leaves: De novo Transcriptome Assembly, Functional Annotation and Development of Genomic Resources
Source: Front Plant Sci. 2017 Feb 2;8:91. doi: 10.3389/fpls.2017.00091 (PMC5288370; doi:10.3389/fpls.2017.00091)

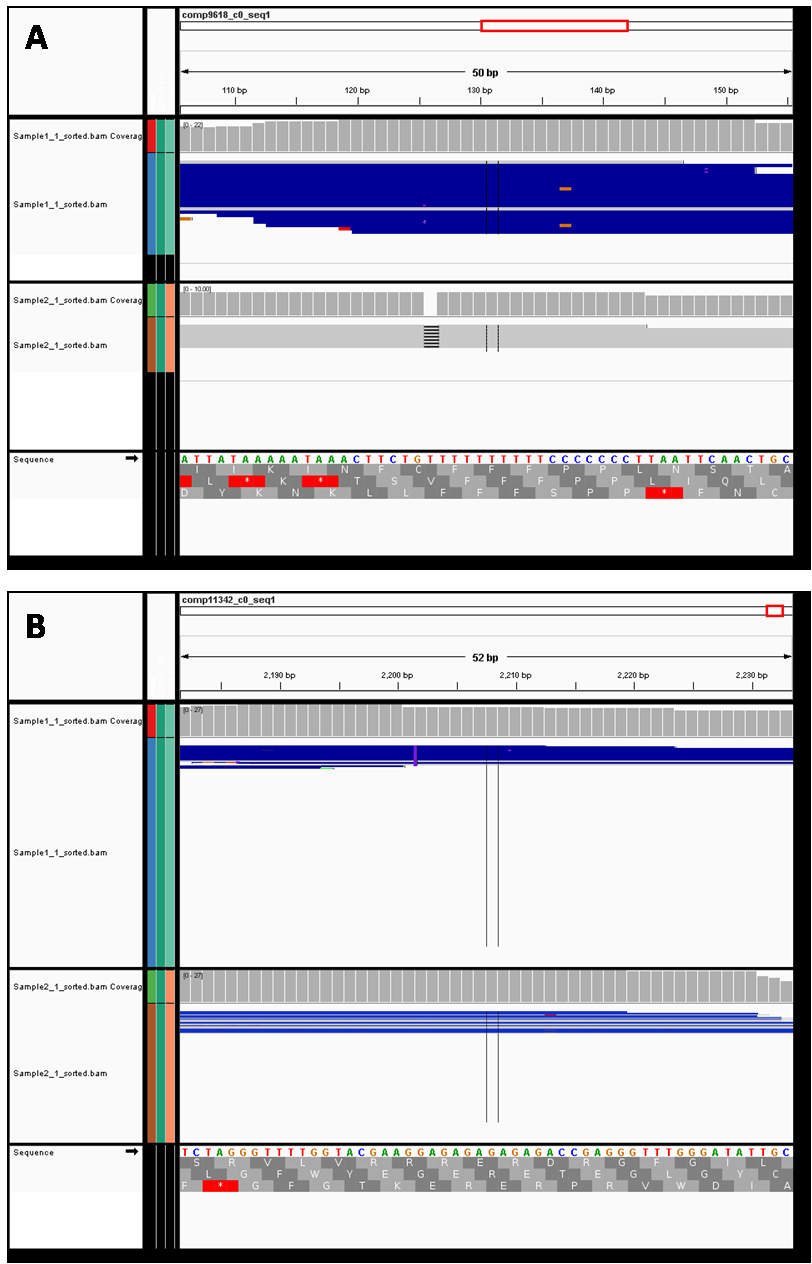

Supplement: Supplementary Figure S1 — The instances of in silico identified polymorphic SSR markers. (A) comp9618_c0_seq1106-155 and (B) comp11342_c0_seq12,182-2,233. [file Image1.tif]
